# Differences in Treating Tobacco Use Across National, State, and Public Hospital System Surveys

**DOI:** 10.5888/pcd15.170575

**Published:** 2018-07-16

**Authors:** Michael D. Celestin, Tekeda Ferguson, Edward C. Ledford, Tung-Sung Tseng, Thomas Carton, Sarah Moody-Thomas

**Affiliations:** 1 Department of Behavioral and Community Health Sciences, School of Public Health, Louisiana State University Health – New Orleans, New Orleans, Louisiana; 2 Department of Epidemiology, School of Public Health, Louisiana State University Health – New Orleans, New Orleans, Louisiana; 3 Louisiana Public Health Institute, New Orleans, Louisiana

## Abstract

The Louisiana Tobacco Control Initiative (TCI), a multidisciplinary program specializing in helping tobacco users quit, assisted health care providers in Louisiana’s public hospitals with integrating evidence-based treatment of tobacco use into clinical practice. Our study compared smoking behavior, provider adherence to the 5 A’s tobacco cessation intervention (ask, advise, assess, assist, and arrange), cessation assistance awareness, quit attempts, and treatment preference among respondents to a TCI survey with a sample of respondents from the National Adult Tobacco Survey (NATS) and a sample from the Louisiana Adult Tobacco Survey (LATS). In 2010, more TCI respondents were asked if they smoked, advised to quit, helped to set a quit date, counseled, and arranged to be contacted for follow-up than respondents to NATS or LATS. Fewer TCI respondents received self-help material or were prescribed medication to assist in quitting than NATS and LATS respondents. In 2010 and 2013, TCI participants reported more quit attempts when 4 or more of the 5 A’s were received. Thus, public health systems can promote treatment of tobacco use.

## Introduction

Smoking causes preventable disabilities, diseases, and death (1). Demographic variables such as sex, insurance status, race, income, and education correlate with smoking (2). Data from the Behavioral Risk Factor Surveillance System (BRFSS) indicate that men, the uninsured, racial/ethnic minorities, people in households with annual incomes of $15,000 or less, and people who were not high school graduates smoke more than their counterparts (3). Of tobacco users, 80% see a health care provider at least once a year, allowing providers to reach large populations of smokers to advise quitting and to assist with access to treatment of tobacco dependence (4).

Clinical practice guidelines for treatment of tobacco use recommend the 5 A’s (ask, advise, assess, assist, arrange) intervention: ask every patient about tobacco use and document status, advise tobacco users to quit, assess user willingness to make a quit attempt, assist users willing to quit by providing or referring for counseling and recommending or prescribing cessation medication, and arrange for follow-up contact to help smokers quit (5). When providers adhere to the protocol, they improve quit rates, health outcomes, and satisfaction with care among tobacco users, and when the 5 A’s are paired with systems changes and increased access to counseling and medication, the intervention produces higher abstinence rates than using any of these approaches alone (6). However, adherence to these strategies remains low (7).

In 2002, the state of Louisiana funded the Louisiana State University Health–New Orleans (LSUH–NO) School of Public Health to create the Louisiana Tobacco Control Initiative (TCI) for the state’s public hospital system operated by the Louisiana State University (LSU) Health Care Services Division and serving predominantly low-income, low-educational level, racial/ethnic minority, and chronically ill populations. In 2003, TCI assisted hospitals with integrating guideline-recommended interventions, including the tobacco 5 A’s intervention, into routine care in outpatient clinics (8). TCI selected the 5 A’s intervention because of its representativeness of core elements of a tobacco intervention and flexibility in provider delivery (6). To monitor 5 A’s performance among providers and to assess patient outcomes, TCI administered a patient survey similar to the existing National Adult Tobacco Survey (NATS) and Louisiana Adult Tobacco Survey (LATS) (9), and the National Health Interview Survey (NHIS) (10). Patients themselves offer the most complete and accurate reflection of the effectiveness of treatment, and their perspectives should be captured systematically and directly (11). Our study compared smoking behavior, provider adherence to the 5 A’s intervention, cessation assistance awareness, quit attempts, and treatment preference as reported by state public hospital patients to the NATS sample in 2010 and the LATS sample of the state’s general population in 2010 and 2013.

## Samples, Measurements, and Analysis

### Samples

For comparisons between state and national trends, we removed the LATS sample from the NATS, creating a subsample of respondents to compare with participants from the remaining 49 states. Therefore, we analyzed 3 data sets: 1) the 2010 NATS without LATS, 2) LATS, and 3) the TCI patient survey. Data in all 3 surveys were self-reported. NATS was not administered in 2013. Because income is a confounder, we included only the subsets of respondents in NATS and LATS who had an annual income of less than $50,000 to compare with the TCI patient population. Details on survey methodology are available (12–14).

**NATS.** NATS is a random-digit–dialed telephone survey of the noninstitutionalized US population aged 18 or older. In 2010, survey professionals administered standardized questionnaires to a sample of 118,581 respondents to collect data on tobacco use, smoking cessation, and tobacco-related treatment. We limited our sample to respondents who visited a health care provider in the past year and who had an annual income of less than $50,000, for a total of 39,563 respondents.

**LATS.** LATS, administered annually, is a stratified, random-digit–dialed telephone survey of noninstitutionalized adults aged 18 or older residing in Louisiana. Like NATS, LATS collects data on tobacco use, smoking cessation, and treatment of tobacco use. LATS consists of questions taken from NATS and questions added by the state. The Louisiana state samples consisted of 6,351 (2010) and 6,403 (2013) respondents. By limiting the samples to respondents who visited a health care provider in the past year and who had an annual income less than $50,000, our final samples consisted of 2,329 respondents for 2010 and 2,084 for 2013.

**TCI patient survey.** The TCI patient survey is an annual survey of patients aged 18 or older cared for by primary care providers in the LSU Health Care Services Division public hospital system. The TCI survey is self-administered in the clinic with cessation coordinators available to assist patients in understanding questions and to verify completion. The TCI survey contained questions similar to those in national surveys (eg, NHIS, NATS, LATS, and BRFSS), and consisted of 3 sections: 1) Tobacco Use, 2) Physician and Health Professional Behavior, and 3) Quit Attempts and Preferred Methods to Quit. The total number surveyed by TCI was 890 (2010) and 1,209 (2013).

### Measurements

Measurement of the 5 A’s followed the methods of King et al (2). TCI used an electronic health record (EHR) system to prompt completion of the 5 A’s–based clinical intervention for a brief intervention (<10 min). The EHR prompted providers to conduct the intervention every 90 days. During the clinic visit, 4 of the 5 A’s could be conducted and documented in the EHR by physicians, nurses, medical assistants, or other health professionals. The EHR transmitted this information to tobacco treatment specialists who arranged for patients who were ready to quit within the next 30 days to receive their selected treatment option.

Among respondents reporting smoking at least 100 cigarettes in their lifetime, those who also reported smoking at least once in the past 30 days were defined as current smokers and those who did not report smoking in the past 30 days were defined as former smokers. Never smokers consisted of respondents who answered no to smoking at least 100 cigarettes in their lifetime. Heavy smokers were defined as those smoking at least 10 or more cigarettes per day. A quit attempt was characterized as a current smoker reporting not smoking for one day or longer in the previous 12 months; quit duration was characterized as the length of time former smokers remained abstinent. For those who reported making a quit attempt, variables were awareness of quit assistance and treatment used the last time respondents tried to quit, including medication and behavioral counseling (individual, group class, or quit-line counseling). Income data were available only for NATS and LATS.

### Statistical analysis

Student *t* tests and χ^2^ analyses determined differences between groups in demographics, smoking behavior, receipt of the 5 A’s, awareness of quit assistance, quit attempts, and treatment use across surveys at the *P* < .05 level. Logistic regression analyses determined differences in receiving 3 or more of the 5 A’s; bivariate regression analyses determined if receiving an increased number of A’s influenced quit attempts, with sex, age, and race as predictor variables. We used Stata 13 statistical software (StataCorp) to apply sampling weights to the NATS sample and to control for clustering of respondents in each sample (by clinic for the TCI sample and by region for the NATS sample).

## Variations Among Respondents to the Three Surveys

### Comparisons of demographics and smoking behavior

We compared sex, age, race, and annual income across the 3 surveys. In 2010 and 2013 more TCI survey respondents identified as women than NATS and LATS respondents (Table 1). However, fewer TCI survey respondents identified as women in 2013 than in 2010. The percentage of TCI respondents aged 18 to 24 declined, from 7% in 2010 to 2% in 2013, and the percentage was lower in this TCI age group than among NATS and LATS respondents for either year. In 2010, the percentage of white TCI respondents was roughly the same (52%) as LATS respondents, but lower than NATS respondents (65%). The percentage of 2013 LATS white respondents increased to 57% from 52% in 2010, while the percentage of 2013 TCI white participants decreased from 52% to 48%. In 2010, more LATS respondents reported annual incomes less than $20,000 than NATS respondents. The number of LATS respondents reporting annual incomes less than $20,000 declined from 2010 to 2013, from 34% to 27%.

**Table 1:** Demographic Characteristics and Smoking Behavior Reported by Participants in Three Tobacco-Related Surveys, Louisiana, 2010 and 2013^a^

Characteristic	2010			2013	
	NATS	LATS	TCI	LATS	TCI
**Total, n**	39,563	2,329	890	2,084	1,209
**Sex**					
Female	57.9^b^	57.7^c^	75.7^d^	56.5^e^	67.7
Male	42.1	42.3	24.3	43.5	32.3
**Age, y**					
18–24	14.1^b^	13.9^c^	6.7^d^	14.5^e^	2.4
25–34	17.6	17.2	11.8	18.1	7.0
35–44	14.9	13.3	18.0	12.4	12.8
45–54	16.0	17.4	23.6	15.1	29.0
55–64	14.1	15.9	24.7	16.5	33.7
≥65	23.3	22.4	15.3	23.3	15.1
**Race**					
White	65.4^b^^,^ f	52.3^g^	51.9	57.1^e^	47.7
Black	14.9	41.6	48.1	33.1	46.0
Other	19.8	6.1	0	9.9	6.3
**Annual income, $**					
<20,000	27.0^f^	34.0^g^	NA	27.1	NA
20,000–29,000	21.3	21.4		20.6	
30,000–39,000	24.7	20.2		27.3	
40,000–49,000	27.1	24.5		25.1	
**Smoker status**					
Never	50.9	50.8^g^	51.1^d^	59.0^e^	43.7
Former	25.0^b^	22.6	19.0^d^	19.3^e^	24.2
Current	24.2^b^	26.6^c^^,^ g	31.7	21.7^e^	32.3
Heavy^h^	61.8	64.9	60.6	54.3	56.6
**Quit duration**					
>3 months	90.8	92.9	88.6	90.1	91.4
>12 months	82.7	84.1^g^	79.6	74.2^e^	87.3

Abbreviations: LATS, Louisiana Adult Tobacco Survey; NA, not available; NATS, National Adult Tobacco Survey; TCI, Tobacco Control Initiative.

a Values are percentages unless otherwise noted.

b *P* < .05, NATS 2010 versus TCI 2010.

c *P* < .05, LATS 2010 versus TCI 2010.

d *P* < .05, TCI 2010 versus TCI 2013.

e *P* < .05, LATS 2013 versus TCI 2013.

f *P* < .05, NATS 2010 versus LATS 2010.

g *P* < .05, LATS 2010 versus LATS 2013.

h Heavy smoker = average ≥10 cigarettes per day.

Smoking status varied among all 3 surveys from 2010 to 2013 (Table 1). The percentage of TCI respondents who reported never smoking in 2010 (51%) declined to 44% in 2013, whereas the percentage of LATS respondents who never smoked increased from 51% in 2010 to 59% in 2013. The percentage of TCI respondents who identified as former smokers increased from 2010 (19%) to 2013 (24%). The percentage of TCI former smokers was lower than NATS and LATS in 2010, but higher than LATS in 2013 (19% LATS vs 24% TCI). In both years, a larger percentage of TCI survey respondents were current smokers than were NATS and LATS respondents. Among LATS respondents, the percentage of current smokers decreased from 2010 (27%) to 2013 (22%); however, rates remained the same for TCI participants (32%). More LATS respondents reported quitting smoking for longer than 12 months in 2010 (84%) than in 2013 (74%). In 2013 more TCI respondents (87%) reported quitting for more than 12 months than LATS respondents (74%).

### Receipt of the 5 A’s

In 2010, more TCI respondents reported being asked if they used tobacco and advised to quit than did NATS and LATS respondents (Table 2). The percentage of TCI respondents who were advised to quit (80%) remained approximately the same from 2010 to 2013; however, the percentage increased for LATS respondents, from 63% in 2010 to 75% in 2013. More TCI respondents (73%) reported being assessed for readiness to quit in 2013 than in 2010 (61%). In 2010, fewer TCI respondents (47%) reported being assisted with quitting than NATS respondents (57%). The percentage of LATS respondents who reported being assisted with quitting declined from 48% in 2010 to 45% in 2013, whereas the percentage of TCI respondents who reported being assisted increased, from 47% in 2010 to 59% in 2013.

**Table 2:** Smoking Cessation Assistance Using 5 A’s (Ask, Advise, Assess, Assist, Arrange) Tobacco Cessation Intervention Reported by Respondents to 3 Surveys, Louisiana, 2010 and 2013^a^

Respondents	2010			2013	
	NATS	LATS	TCI	LATS	TCI
**Total, n**	7,347	439	252	347	360
**No. of 5 A components offered, mean (SD)**	2.50 (1.51)	2.34 (1.50)	2.52 (1.47)	2.43 (1.39)	2.84 (1.34)
**Component offered**					
*Asked* if smokes	88.2^b^	85.6^c^	93.8	91.5	95.5
*Advised* to quit	67.1^b^	63.1^c^^,^ d	80.3	75.0	80.7
*Assessed* willingness to quit	65.7	65.2	61.3^e^	64.8	73.4
*Assisted* in quitting	56.5^b^	48.1^d^	46.5^e^	44.5	58.8
Prescribed medication	63.6^b^	65.7^c^	41.9	68.3^f^	35.0
Helped with setting quit date	15.7^b^	11.9^c^	46.5	25.5	37.1
Provided counseling	47.5^b^	56.9^c^^,^ d	80.2	79.2	68.6
Provided smoking cessation materials	62.6^b^	56.0^c^^,^ d	36.1	78.4^f^	37.1
*Arranged* for follow-up	17.7^b^	13.4^c^	32.6^e^	19.3	17.9
**Patient aware assistance available**	48.2^g^	36.8^c^	48.2^e^	47.1^f^	62.1
**Patient made quit attempt**	47.5^b^	51.6	54.8	55.7	52.9
**Type of treatment provided**					
Medication	41.1^b^	34.8^c^	18.0	36.5^f^	15.7
Behavioral counseling	16.9	18.4	22.7^e^	10.3	8.5
Group class counseling	8.0^b^	9.2	14.6	1.9	7.2
Quit-line counseling	6.7	7.0	3.5	4.3	2.0
Individual counseling	8.3^g^	3.6^c^	9.5^e^	8.6^f^	0.7

Abbreviations: LATS, Louisiana Adult Tobacco Survey; NATS, National Adult Tobacco Survey; TCI, Tobacco Control Initiative.

a Values are percentages unless otherwise noted.

b *P* < .05, NATS 2010 versus TCI 2010.

c *P* < .05, LATS 2010 versus TCI 2010.

d *P* < .05, LATS 2010 versus LATS 2013.

e *P* < .05, TCI 2010 versus TCI 2013.

f *P* < .05, LATS 2013 versus TCI 2013.

g *P* < .05, NATS 2010 versus LATS 2010.

Fewer TCI respondents were prescribed medication and provided with self-help materials than NATS and LATS respondents in both study years (Table 2). In 2010 more TCI respondents were assisted with setting a quit date and provided with counseling compared with NATS and LATS respondents. The percentage of LATS respondents who received counseling and self-help materials increased significantly, from 57% in 2010 to 79% in 2013. Finally, more 2010 TCI respondents (33%) had follow-up contact arranged than NATS (18%) and LATS (13%) respondents. However, that percentage decreased significantly for TCI respondents in 2013, to 18%.

### Awareness of quit assistance, quit attempts, and treatment provided

We assessed the 3 survey samples for respondents’ awareness of the availability of assistance in quitting smoking, the number of quit attempts they made, and the types of treatment provided to them (Table 2). In 2010, the number of TCI respondents who were aware that assistance with quitting was available was the same as NATS respondents (48%) and higher than LATS respondents (37%). Those numbers increased for both TCI (62%) and LATS (47%) in 2013. In 2010, more TCI survey respondents made a quit attempt in the previous 12 months than either NATS or LATS respondents. Fewer TCI respondents reported using medication to help quit smoking in their latest quit attempt than NATS or LATS participants in either survey year. Use of behavioral counseling declined for TCI survey participants, from 23% in 2010 to 9% in 2013. Use of group or individual counseling varied across the 3 survey groups, and a relatively small percentage of respondents used either. In 2010 more TCI respondents used group or individual counseling than NATS or LATS respondents, but percentages changed significantly in 2013, declining for TCI respondents and increasing for LATS respondents.

## Increasing the Odds of Quitting

We examined the odds of survey respondents receiving any of the components of the 5 A intervention and making a quit attempt. In 2010 NATS respondents who received 3 or more of the 5 A’s compared with none of the 5 A’s (odds ratio [OR] = 1.74; 95% confidence interval [CI] 1.19–2.55) had increased odds of making a quit attempt (Table 3). In 2010 and 2013, TCI respondents had increased odds of making a quit attempt by receiving 4 or more of the 5 A’s compared with none; in 2010, they had more than 3 times greater odds of making a quit attempt by receiving 4 or more of the 5 A’s compared with none (OR= 3.32; 95% CI, 1.06–10.46). The largest odds ratio for making a quit attempt was among 2013 TCI survey respondents who received 4 or more of the 5 A’s compared with none (OR= 4.28; 95% CI, 1.25–14.63).

**Table 3:** Adjusted^a^ Odds Ratios of Having Received Any Component of the 5 A's Tobacco Cessation Intervention and a Quit Attempt, Respondents to 3 Surveys, Louisiana, 2010 and 2013

No. of 5 A’s	2010			2013	
	NATS	LATS	TCI	LATS	TCI
**0**	1.0 [Reference]	1.0 [Reference]	1.0 [Reference]	1.0 [Reference]	1.0 [Reference]
**1**	.99 (.68–1.44)	1.52 (.47–4.91)	1.84 (.55–6.16)	1.38 (.33–5.78)	2.19 (.60–8.05)
**2**	1.43 (.99–2.05)	1.55 (.50–4.80)	2.94 (.92–9.37)	.92 (.23–3.74)	1.60 (.43–5.96)
**3**	1.74 (1.19–2.55)	2.36 (.67–8.39)	3.11 (.94–10.29)	1.60 (.42–6.08)	2.84 (.82–9.88)
**≥4**	1.83 (1.31–2.54)	2.43 (.82–7.18)	3.32 (1.06–10.46)	1.73 (.46–6.58)	4.28 (1.25–14.63)

Abbreviations: LATS, Louisiana Adult Tobacco Survey; NATS, National Adult Tobacco Survey; TCI, Tobacco Control Initiative.

a Models adjusted for age, sex, and race. Values are adjusted odds ratios.

## Summary

Our purpose was to compare smoking behavior, receipt of 5 A’s tobacco intervention, awareness of quit assistance, quit attempts, and treatment preference as reported by public hospital patients in the TCI survey with NATS and LATS respondents. First, TCI survey respondents reported higher smoking rates than state and national respondents. Consistent with current literature, smoking is associated with low income worldwide and across various population subgroups (15). Second, TCI survey participants used medication during their most recent quit attempt less often than state and national survey participants. Because cessation interventions, including medication, are effective for all sociodemographic groups (5), future studies should examine why low-income populations do not select medication as a treatment option and should test interventions to increase its use. Third, more TCI survey respondents used group behavioral counseling during their most recent quit attempt than NATS and LATS respondents. Since 2003, TCI has emphasized clinic referral to intensive behavioral counseling by a tobacco treatment specialist who serves as a navigator to external cessation services.

Fourth, in 2010 and 2013, more TCI survey respondents than NATS and LATS respondents reported being asked if they used tobacco and advised to quit, assisted with setting a quit date and provider counseling, and arranged follow-up contact. Additionally, more TCI respondents made a quit attempt and used group and individual counseling than NATS and LATS respondents. Engagement by providers, quit attempts, and sustained quits increase when health systems integrate guideline-recommended strategies (5). This includes changes at the system, clinic, and patient levels, such as ongoing provider education, marketing, and outreach to patients, which may account for the greater awareness of assistance among TCI survey respondents.

The strengths of this study include the use of identical survey questions, survey administration in the same year for all 3 instruments, and consistent use of these methodologies across multiple years. The study has 6 limitations: 1) Self-reported data may be influenced by differences in recall resulting from methods of survey administration procedures (ie, self-administered vs telephone vs immediately following a clinic visit) and by recall bias. 2) LATS and NATS respondents were included if they reported having visited a doctor in the past year, a time frame different from the TCI sample, which was collected during the clinic visit. 3) The study lacks details of any other 5 A’s intervention to which subjects, other than TCI survey participants, were potentially exposed. 4) The use of low-income respondents from LATS is problematic because it likely draws from the same population served by the public hospital system studied in the TCI survey. Unfortunately, we cannot distinguish or ensure that any of the respondents are in mutually exclusive groups. 5) Results in Table 2 are limited because we did not adjust to account for age, sex, and race differences. 6) Results in Table 3 show estimates with large confidence intervals resulting from small sample sizes.

## Public Health Implications

Surveillance and effective treatment of tobacco use are needed. Clinical practice guidelines allow standardization of approaches to tobacco treatment, and patient-reported outcomes allow tailoring of health care guided by these reports. Our study demonstrates the potential for health care delivery systems to create standardized tobacco surveillance across instruments, sites, and credentialing agencies and to compare patient-reported data to national surveillance data. These results can be used in the development of models that improve delivery of treatment of tobacco dependence, acceptability of care, and decision making for individual options for treating tobacco use.
